# Does a patient’s physical activity predict recovery from an episode of acute low back pain? A prospective cohort study

**DOI:** 10.1186/1471-2474-14-126

**Published:** 2013-04-05

**Authors:** Paul Hendrick, Stephan Milosavljevic, Leigh Hale, Deirdre A Hurley, Suzanne M McDonough, Peter Herbison, G David Baxter

**Affiliations:** 1 Division of Physiotherapy Education, University of Nottingham, Hucknall Road, Nottingham NG5 1PB, UK; 2 Centre for Physiotherapy Research, School of Physiotherapy, University of Otago, Dunedin, New Zealand; 3 School of Public Health, Physiotherapy and Population Science, College of Life Sciences, University College Dublin, Belfield, Ireland; 4 Faculty of Life and Health Sciences, University of Ulster, Newtownabbey, Northern Ireland; 5 Department of Preventive and Social Medicine, Dunedin School of Medicine, University of Otago, Dunedin, New Zealand

**Keywords:** Physical activity, Acute low back pain, Recovery, Predictor, Cohort

## Abstract

**Background:**

Advice to remain active and normalisation of activity are commonly prescribed in the management of low back pain (LBP). However, no research has assessed whether objective measurements of physical activity predict outcome and recovery in acute low back pain.

**Method:**

The aims of this study were to assess the predictive relationship between activity and disability at 3 months in a sub-acute LBP population. This prospective cohort study recruited 101 consenting patients with sub-acute LBP (< 6 weeks) who completed the Roland Morris Disability Questionnaire (RMDQ), the Visual Analogue Scale, and resumption of full ‘normal’ activity question (Y/N), at baseline and 3 months. Physical activity was measured for 7 days at both baseline and at 3 months with an RT3 accelerometer and a recall questionnaire.

**Results:**

Observed and self-reported measures of physical activity at baseline and change in activity from baseline to 3 months were not independent predictors of RMDQ (p > 0.05) or RMDQ change (p > 0.05) over 3 months. A self-report of a return to full ‘normal’ activities was significantly associated with greater RMDQ change score at 3 months (p < 0.001). Paired t-tests found no significant change in activity levels measured with the RT3 (p = 0.57) or the recall questionnaire (p = 0.38) from baseline to 3 months.

**Conclusions:**

These results question the predictive role of physical activity in LBP recovery, and the assumption that activity levels change as LBP symptoms resolve. The importance of a patient’s perception of activity limitation in recovery from acute LBP was also highlighted.

**Trial registration:**

Clinical Trial Registration Number, ACTRN12609000282280

## Background

Maintenance and normalization of activity and an early return to work are key clinical guideline recommendations in the management of acute low back pain (LBP) [1,2]. Others have hypothesised that deconditioning results from prolonged LBP [3], as part of which disability and pain exacerbate patients’ beliefs to refrain from physical activity (PA) [4]. Hasenbring [5] previously described the potential behavioural adaptations to LBP, with fear avoidance being the potential mediator of decreased activity in LBP populations [6]. However deconditioning within chronic LBP populations has been questioned [7], as individuals with chronic LBP have demonstrated similar (objective based) functional assessments [8], and activity levels, as matched controls [6,9].

Few studies have objectively measured PA in a prospective, longitudinal design to assess the influence of type and level of physical activity on long-term outcomes in this population. Investigation using an RT3 accelerometer to measure activity found no relationship between activity change and disability at the 1-year point in a sub-acute LBP population [10]. A number of studies have prospectively assessed the relationship of activity to recovery in LBP populations employing questionnaire-based activity recall instruments. One such study found that the cross sectional and longitudinal odds ratio (OR) of back disability were significantly reduced in those with the highest levels of reported leisure time PA levels [11]. Other studies have found no relationship between levels and types of activity in various LBP cohorts and measures of LBP disability, pain [12,13] or return to work [14].

The factors associated with the transition from acute to chronic pain are a focus in the primary care management of acute LBP [15]. Although there is a consensus in International guidelines on activity ‘re-activation’ during an episode of non-specific LBP [16], there is little known on whether the types and levels of activity of patients with LBP affect the course of such episode of LBP. No studies have assessed whether activity levels change over time in an acute LBP population, and whether such changes predict recovery. It can be hypothesised that a positive and significant change in activity over a course of LBP will be a positive predictor of recovery. Therefore the primary aims of this study were to therefore to investigate:

1. whether people with acute LBP change their activity levels over a 3 month period

2. whether people’s activity levels (and potential change in activity levels) are predictors of disability at 3 months from an episode of acute LBP

## Methods

### Study design

A prospective cohort study, conducted from March 2008 to April 2009, measured observed change in physical activity in patients with acute LBP. The main outcome measure used for calculation of sample size was change in the Roland Morris Disability Questionnaire (RMDQ) score from baseline to 3 months, and calculated for 80% power with a two-sided test and significance set at p = 0.05. Based upon a clinically meaningful change of 4 points (and SD = 5.4) in the RMDQ [17], and assuming unequal group sizes between those whose PA levels change by 4 points or greater, versus those whose PA levels change minimally over a 3 month period, required a sample size of 65 participants. An estimated attrition rate of 40% over the two time points increased the required sample size to 100 participants.

### Participant recruitment

Following ethical approval from the New Zealand Lower South Regional Ethics Committee (LRS/07/11/043), 101 participants were recruited by public advertising within physiotherapy clinics (n = 77) and general practice (GP) clinics (n = 16), as well as by newspaper advertisement (n = 8). Eligible participants had an episode of LBP of 6 weeks or less, preceded by a minimum period of 3 months during which participants had not sought treatment for LBP. Participants were aged between 18 and 65 years, and had no other pre-existing conditions which limited their mobility. All participants were required to provide written informed consent to participate in this study and were given a $10 voucher as a measure of thanks and recompense for their participation. All participants were required to be receiving physiotherapy treatment for the current episode of acute LBP to be eligible to participate in the study. Physiotherapy treatment was administered by their treating physiotherapist and they were free to take a pragmatic approach with regards to the types and duration of treatment the patients received. The principal investigator (PI) screened each participant for the following exclusion criteria: serious or systemic spinal pathologies including persistent or progressive neurological deficit, intractable pain, spinal surgery, or inflammatory disorders.

### Physical activity measurement

The RT3 accelerometer (Stayhealthy, Inc., Monrovia, California) is a small, pocket sized, portable triaxial activity monitor. The RT3 monitor is a triaxial accelerometer used to measure physical activity in free living and is approximately the size of a personal pager (71 × 56 × 28mm, weight 65g, with one AAA sized battery). When firmly attached to clothing or a belt at the waist, it measures the acceleration of bodily movement in three directions, vertical (X), anterioposterior (Y) and mediolateral (Z), converting this movement into raw counts. Vector magnitude (VM) is the square root of the sum of the squared counts in each direction and is utilized as a measure of accelerometry output representing PA. The RT3 monitor can store data for up to seven days while simultaneously recording from all three axes at one-minute intervals. Proprietary interface software allows the download of data to an appropriate computer database. The software converts activity counts into kcals/minute from a physiological regression equation developed by the manufacturer; helping to calculate both activity related energy expenditure (EE) and total EE.

The RT3 is capable of collecting and storing data in 1-minute epochs for 21 days, and has no external controls that allow the person being tested to manipulate or change recorded data. The RT3 is therefore potentially suitable for long term tracking of PA data in the home environment [18]. The RT3 demonstrates high levels of intra-monitor reliability [19], although levels of inter-monitor variability are more variable [20]. To evaluate the technical performance of the nine RT3 accelerometers in this study for field use, each monitor was subjected to specific vibration testing along each sensitive axis in isolation. Inter-instrument CV across all monitors on each of the 3 axes ranged from 10.8 – 35.7, and the intra-class correlation (ICC) for intra-axis reliability ranged from 0.98-0.99 on the 3 axes. Due to the variable inter-monitor reliability, each participant was given the same monitor on the repeat testing measurement. The RT3 tri-axial accelerometer accumulated vector magnitude (VM) activity counts for each one-minute epoch over the 7 days of monitoring.

All participants were recruited through Physiotherapy clinics in the Dunedin region and all were currently receiving Physiotherapy treatment for their LBP. For the purposes of this study, the form of treatment was not specified and Physiotherapists were advised to educate their patients around PA according to standard accepted guidelines [1]. The RT3 was attached over the right hip at the level of the iliac crest for seven days in a harness. Participants wore the RT3 during waking hours (except when performing activities which might cause it to become wet, such as bathing or swimming) for 7 consecutive days. Participants also completed an activity diary [21] which detailed their predominant daily activities during each hour, their sleep times, and also noted removal of RT3 and reasons for such removal. Participants were contacted twice during the week to improve compliance in wearing the RT3, recording activity in the activity dairy, and to address any problems the participants were having with either the RT3 or in using the activity diary. After seven days the participant met with the PI at either the School of Physiotherapy or the participant’s home/work. RT3 data was downloaded to a portable computer by the PI and the daily activity diary collected.

A 7-day recall questionnaire (7D-PAR) [22] was completed by interview at the end of each week of activity assessment. This questionnaire is accepted as providing reasonable validity as a measure of free living energy expenditure (EE) within various populations [23,24]. Participants were asked to recall the amount of time spent in sleep, moderate, hard, and very hard activities during weekdays and weekend days of the past 7 days. This allowed an estimate of the average total daily energy expenditure (TDEE) and physical activity energy expenditure (PAEE) for each participant, as well as an estimate of the time spent in each of the activities indicated. Participants also completed an activity diary [25] which detailed their predominant daily activities during each hour, their sleep times, and also noted removal of RT3 and reasons for such removal.

Habitual activity levels prior to the onset of LBP were measured with the Baecke Physical Activity Questionnaire (BPAQ), which gathers information on “usual” activity levels over the previous year. The BPAQ demonstrates good repeatability and relative validity in free living populations [26], and high test-re-test reliability in low back pain populations [27]. The questionnaire is divided into three sections (work, sports, and leisure), which are individually scored [28]. The questionnaire was worded such that participants were asked about activity levels over the previous year prior to the onset of LBP.

### Low back pain measurements

Participants completed a number of validated LBP outcome measures at baseline and at 3 months. The primary outcome measure was a change in RMDQ. The 24 item RMDQ has been shown to be a valid measure of LBP disability and sensitive measure of change in functional disability in LBP populations [28]. This self-administered questionnaire consists of 24 items which refer to limitations of daily activities as a result of LBP. Total score ranges from 0 to 24 (higher scores indicate more disability). Secondary outcomes were pain intensity, measured with the Visual Analogue Scale (VAS) which is a scaled measurement of pain (0 – 100) in which the participant was asked to rate the average level of pain over the past 7 days. This tool has been shown to be a valid, reliable and appropriate tool for use in clinical practice [29], and sensitive to change [30]. A specific activity question, developed for this study, asked participants *Do you consider that you have returned to full “normal” activities since this current episode of low back painn* (Yes/No).

Participants also completed the Fear-Avoidance Beliefs Questionnaire (FABQ), and the 12-item General Health Questionnaire (GHQ12). The FABQ has been shown to be a reliable measure of pain-related fear in acute LBP populations [31] and demonstrates strong predictive validity for functional disability in both acute [32] and chronic LBP populations [33]. The GHQ12 is a validated measure of psychological distress in the general population [34] as well as LBP populations [35]. Prospective research has shown that GHQ12 scores can predict future episodes of LBP [36]. Main occupation was also recorded and dichotomised into manual (involving manual lifting, heavy labour, or regular bouts of physical activity or exertion) or non-manual occupation. Each participant’s height and weight were also recorded to calculate their BMI.

### Procedure

Full details of the methodology are available in Hendrick et al. [37]. In brief, participants were recruited from physiotherapy practices within the Dunedin and Otago region (approx. population of 250,000). The principal investigator (PI) met with the participant at the physiotherapy practice or at the participant’s home or work. Each participant completed the baseline LBP outcome measures and the FABQ, GHQ12, and BPAQ. The participant’s age, height, weight, age, occupation, and ethnicity were recorded, and the participant instructed to wear the RT3 over the right pelvis during waking hours, and only to remove it for sleep and water-based activities. Participants were also asked to record their main activity for each hour in an activity diary, and to note the time and reason for removing the RT3. In order to improve compliance, participants were contacted twice during the week that they wore the RT3. Participants were instructed to continue their “normal” activity levels; they did not receive specific physical training during this week nor given any specific instructions regarding activities. At the completion of the week of monitoring, participants met the PI, the RT3 was removed and the data downloaded to a computer and checked to ensure that measurement protocols were met. To be included in the analyses, each participant was required to have recorded a minimum of 10 hours of RT3 data on five or more days, including one weekend day [38]. Sleep times were also ascertained, the data scanned and possible RT3 malfunctions identified. Data were then scanned for non-worn periods (> 20 minutes of zero VM activity) and such data were set to an electronic file labelled as ‘missing’ [39]. Participants returned the activity diary and completed the 7D-PAR under the guidance of the PI. Each participant repeated the activity monitoring procedure and the LBP outcome measures as per baseline at 12 weeks following the first measurement point.

### Analyses

The main outcome variables were RMDQ at 3 months and change in RMDQ from baseline to 3 months. The change score was calculated by subtracting each participant’s 3 month RMDQ score from their baseline RMDQ score. The sum of RT3 activity counts for each day was calculated as well as the total number of hours of activity data collected on each day. The RT3 activity levels were calculated from the estimated wear periods and total weekly activity counts were then divided by the total number of hours worn. The RT3 score was expressed as VM counts/hour/week. Change in RT3 activity was calculated as VM counts/hour/week (VM/hr/wk) at 3 months – VM/hr/wk at baseline. The SD of RT3 VM/hr/wk was calculated at the two time points as a measure of variability in PA [40]. High RT3 VM/hr/wk change was determined as greater than the 75th percentile change in RT3 VM/hr/wk. Daily physical activity energy expenditure (PAEE) from the 7D-PAR (kcal/kg/day) was calculated as the average number of hours in each activity multiplied by the metabolic equivalent (MET) value assigned to the activity category (light = 1.5, moderate = 4, hard = 6, very hard = 10) [41]. Change in PAEE was calculated as the average daily PAEE at 3 months – average daily PAEE at baseline. Paired t-tests and Pearson correlations analysed the difference and the correlation between the groups of parametric scale variables respectively. Comparisons of the binary variable “returned to ‘normal’ activities” at the two time points were made using McNemar’s test. Kendall tau correlations assessed the correlation between the dichotomous variables (return to ‘normal’ activities) and RT3 VM/hr/wk at baseline and 3 months

Simple linear regression was used to assess the unadjusted relationship between PA and PA change and the two main outcome measures ΔRMDQ and RMDQ at 3 months. The explanatory variables included activity levels at baseline (measured with the RT3 and 7d-PAR), change in PA (baseline to 3 months with the RT3 and 7d-PAR), age, sex, occupation, BMI, as well as baseline levels of pain, depression, anxiety, emotional distress and fear avoidance (GHQ12 and FABQ) and activity levels prior to the onset of LBP (Baecke work, sport and leisure scores). A separate analysis was performed to investigate whether change in activity in participants with a low activity at baseline as recorded by the RT3 and 7D-PAR predicted RMDQ change. For the purposes of this research a low activity was defined as below the mean value for both RT3 VM/hr/week and PAEE (kcals/kg). Two types of adjusted analyses were carried out. The first adjusted for all variables whose p-value was < 0.1 in the unadjusted analyses. The second began with these variables and performed a backwards selection multiple linear regression model, which forced RT3 activity level at baseline and change in PAEE as recorded by the RT3 to be included in the model. Assumptions for the regression model were tested by investigating the normalized distribution of residuals and also testing for collinearity and homoscedasticity of the data. All analyses used SPSS software version 14.0 (SPSS Inc., Chicago, Ill).

## Results

Although 101 patients took part in the baseline component of the study, there were only 83 complete RT3 data sets at both baseline and 3 months, and only 90 participants with complete data from the 7D-PAR. Reasons for loss of data included participant drop out at baseline and 3 months (n = 9), RT3 malfunction (n = 6), and RT3 loss (n = 3). The total estimated hours of data loss due to RT3 removal during waking hours was 188.7 hours at baseline and 200.5 hours at 3 months. Table 1 shows the demographic features of the recruited population (n =101). Ethnicity was also recorded with 86% nominating themselves as white NZ European, and 8% Maori or Pacific Islander. Of the 67 workers only 5 reported being currently off work due to their LBP. Table 2 lists fear avoidance scores and PA levels prior to the onset of LBP as well as baseline anxiety and depression scores (GHQ12).

**Table 1:** Baseline demographic measurements

**Baseline descriptive statistics (N = 101)**	**Mean (SD)**
Age	37.8 (14.6)
BMI female (n = 51)	24.7 (4.8)
BMI male (n = 50)	27.6 (4.4)
	n (%)
female	51 (50.5)
male	50 (49.5)
sedentary occupation	24 (23.8)
manual occupation	44 (43.6)
student	25 (24.8)
not working	8 (7.8)

**Table 2:** Baseline measures of fear avoidance, activity and psychological distress

**Baseline Measures**	**Range**	**Mean (SD)**
*FABQpa	0 - 24	14.5 (5.4)
*FABQw	0 - 39	15.8 (9.3)
Baecke Work Index	1.4 – 4.1	2.8 (0.6)
Baecke Sport index	0.8 – 5.8	2.5 (1.1)
Baecke Leisure Time Index	1.8 – 4.5	2.9 (0.5)
αGHQ12	4 - 23	11.8 (4.2)

FABQpa: Fear avoidance beliefs score for physical activity; FABQw: Fear avoidance beliefs score for work: GHQ12 The 12-item General Health Questionnaire.

*Higher numbers indicate greater levels of fear avoidance.

αHigher numbers indicate lower levels of overall self-reported quality of life.

Table 3 records the baseline measures and main LBP outcome measures at 3 months. Baseline measures show moderate levels of disability, with a significant percentage reporting that they had not returned to ‘normal’ activities. At 3 months there was a significant and clinically meaningful change in both the primary and secondary outcome measures [42].

**Table 3:** Comparison of baseline and 3 month outcomes

	**Baseline Mean (SD) (n = 91)**	**3 months Mean (SD) (n = 83)**	**Mean Difference (95% CI)**	^**a **^**P value**
RMDQ score	8.1 (3.8)	1.7 (2.9)	6.1 (5.2 – 7.1)	< .0001
VAS score	57.4 (19.7)	15.2 (19.6)	42.8 (37.4 – 8.2)	< .0001
	N (%)	N (%)		^b^ P value
Return to “normal” activities	23 (22.8)	69 (68.4)		< .0001

^a^Paired t-tests.

^b^McNemar’s test.

### Activity change from baseline to 3 months

Measures of PA at baseline and 3 months are shown in Table 4 for the 83 data sets with complete RT3 data and for the 90 participants with complete data from the 7D-PAR. There were no significant differences in (1) the activity levels recorded with either the RT3 or the 7D-PAR from baseline to 3 months; (2) the amount of hours recorded as moderate, hard, and very hard, from the 7D-PAR from baseline to 3 months. The only difference in baseline measures noted between those lost to follow-up and those with complete data was a greater percentage of those with complete RT3 data having not returned to full ‘normal’ activities at baseline (p < 0.05) (data not shown).

**Table 4:** Comparison of physical activity measures at baseline and 3 months

	**Baseline data Mean (SD)**	**3 month data Mean (SD)**	**Mean Difference (95% CI)**	^**a**^**P value**
RT3 VM/hr/wk	24871.6 (11118)	25410.3 (12388)	538.7 (−23910 - 1322)	0.57
Daily PAEE kcals/kg (7D-PAR)	14.5 (5.7)	15.1 (7.3)	0.6 (−1.8 - 0.7)	0.38
Moderate hours/day	1.6 (1.2)	1.5 (1.7)	0.1 (−0.3 - 0.5)	0.52
Hard hours/day	0.4 (1.0)	0.5 (1.1)	0.1 (−0.2 - 0.1)	0.55
Very hard hours/day	0.1 (0.2)	0.1 (1.7)	0.06 (−0.06 – 0.02)	0.24

^a^ Paired t-tests.

### Predictors of RMDQ at 3 months

In univariate analyses none of the measures of activity at baseline or change in activity from baseline to 3 months predicted RMDQ score at 3 months (Table 5). Significant variables in univariate analyses included in the multiple linear regression model are presented in Table 6. Increasing age was found to be the only variable which predicted RMDQ at 3 months; all other variables were not significant predictors of RMDQ score at 3 months.

**Table 5:** Univariate analyses of physical activity measures as predictors of RMDQ at 3 months

**Parameter**	**β (95% CI)**	**Sig (p value)**
Baseline RT3 VM/hr/wk	0.000 (0.000 – 0.000)	0.20
Baseline PAEE kcals/kg	0.005 (−0.15 – 0.026)	0.59
Report of a return to full activities at baseline	0.071 (−1.144 – 1.54)	0.92
Change in RT3 VM/r/wk from baseline to 3 months	0.00 (0.00 – 0.00)	0.33
*RT3VM_change in low RT3 VM/hr group at baseline	0.004 (0.006 – 0.000)	0.62
Change in PAEE kcals/kg from baseline to 3 months	0.046 (−0.096 – 0.188)	0.52
**PAEE kcals/kg change (7D-PAR) in low activity group at baseline	0.009 (− 0.27 -0.05)	0.63

*Low RT3 VM/r/wk group defined as below the mean RT3 VM/hr/wk at baseline.

**Low PAEE group defined as below mean PAEE kcals/kg at baseline.

**Table 6:** Multiple linear regression analyses of significant predictors in univariate analyses measures of RMDQ score at 3 months

**Parameter**	**β (95% CI)**	**Sig (p value)**
FABQPA	0.091 (−.030 - 0.135)	0.14
GHQ12	0.053 (−.123 - 0.581)	0.58
Age	0.062 (−0.002 – 0.120)	0.05
RT3 VM change from baseline to 3 months	0.000 (0.000 – 0.000)	0.33
Baseline RT3 VM/hr/wk	0.000 (0.000 – 0.000)	0.36

Model adjusted for sex, occupation, BMI, as well as baseline levels of pain, depression, anxiety, emotional distress and fear avoidance (GHQ12 and FABQW, FABQPA) and activity levels prior to the onset of LBP (Baecke work, sport and leisure scores).

### Predictors of RMDQ change from baseline to 3 months

Multiple linear regression analyses including all variables with a p value < 0.1 from the unadjusted analyses and measures of PA change from the RT3 and 7D-PAR are presented in Table 7. The variable associated with a change in RMDQ was the patient’s report of a return to full ‘normal’ activities at the 3 month point. All measures of PA measurement were not predictive of RMDQ change in either univariate or multivariate analyses.

**Table 7:** Multiple linear regression analyses of physical activity measures against RMDQ change

**Predictor**	**β (95% CI)**	**p-value**
RT3 VM/hr/wk change	0.00 (0.00 - 0.00)	0.81
*RT3VM change (low RT3 VM/hr group)	0.00 (0.00 - 0.00)	0.89
PAEE kcals/kg change (7D-PAR)	−0.01 (−0.41 - 0.02)	0.45
**PAEE kcals/kg change (7D-PAR)	0.31 (−0.08 – 0.18)	0.27
Self –report of return to normal activities at 3 months	−3.14 (−4.64 – 1.65)	< 0.001

β Regression coefficients adjusted for PA measures and age, BMI, occupation, activity levels prior to the onset of LBP, fear avoidance, levels of anxiety, depression and baseline RMDQ and pain levels.

*Low RT3 VM/r/wk group defined as below the mean RT3 VM/hr/wk at baseline.

**Low PAEE group defined as below mean PAEE kcals/kg at baseline.

**Figure 1:**
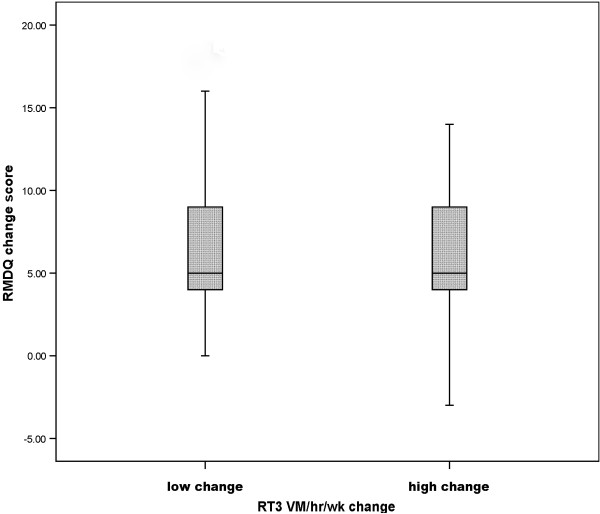
RMDQ change score in groups with low and high RT3 VM/hr/wk change from baseline to 3 months.

Figure 1 shows the RMDQ change score for those with a dichotomized (high/low) RT3 VM/hr/wk change score from baseline to 3 months. The figure demonstrates that there was no difference in the RMDQ change score between the two groups (high and low RT3 change score)

Post-hoc analyses (presented below) were carried out in order to examine for potential correlations between baseline activity measures and disability and also measured activity levels and self-report in an attempt to explain and better understand the observed results.

There was no significant correlation between the observed measures of activity (RT3 VM/hr/wk) at baseline and RMDQ and VAS scores at baseline (Table 8).

**Table 8:** Pearson Correlations between baseline measures of physical activity and baseline RMDQ and VAS scores

	**Baseline RMDQ (p value)**	**Baseline VAS Score (p value)**
Baseline PAEE kcals/kg	−0.19 (0.06)	−0.07 (0.49)
Baseline RT3 VM/hr/wk	−0.14 (0.186)	−0.03 (0.77)

There was no significant correlation between the observed measures of activity (RT3 VM/hr/wk) at baseline and 3 months, and the participant’s report of a return to full ‘normal’ activities at these two time points (Table 9).

**Table 9:** Correlations between RT3 VM/hr/wk and patient’s report of a return to full normal activities at baseline and 3 months

**Kendall’s tau_b correlations**	**Patient report of a return to ‘normal’ activities (p value)**	
Activity measurements	baseline	3 months
Baseline RT3 VM/hr/wk	0.16 (0.06)	-
3 month RT3 VM/hr/wk	-	0.09 (0.29)
Change in RT3 VM/hr/wk	−0.02 (0.82)	−0.06 (0.47)

## Discussion

This study investigated the predictive relationships between observed and self-reported measures of physical activity and LBP disability at 3 months. We found that PA measures at baseline and change in PA as measured by either the RT3 or a recall questionnaire did not predict either RMDQ score at 3 months or change in RMDQ from baseline to 3 months. There were no differences in levels, reported types, intensities of activity at either baseline or at 3 months in this sample of patients with LBP. None of the measures of activity predicted RMDQ at 3 months or change in RMDQ in univariate or multivariate modelling. Increasing age was the only predictor of RMDQ score at 3 months in the multivariate model, and the report of a return to full ‘normal’ activities at 3 months was associated with a greater RMDQ change score from baseline to 3 months.

There have been no previous investigations of the relationship between activity levels and disability within an acute LBP population over this time frame; however these results are consistent with a previous study which found no significant differences in RT3 VM counts at 1 year between recovered and non-recovered LBP participants [10]. A recent cross-sectional study within a chronic LBP population found observably measured activity fluctuation was a significant contributor to disability [39]; however, this study found no change in the variability of activity levels (SD of RT3 VM/hr/wk) between baseline and 3 months, and no predictive relationship to RMDQ at 3 months or change in RMDQ. Differences in these results may relate to differences in patient populations, activity measurement and in particular point of measurement between the two studies.

No previous study has investigated whether activity levels change in an ALBP population over this timeframe. Although there was no change in the types or levels of activity reported by participants from baseline to 3 months, there was a significant change in all outcome measures, with 84% having an RMDQ score < 4 at the 3 month time point. A previous study employing an observed measure of activity reported that PA increased over a 1 year follow-up for both chronic and recovered LBP patients [10]. Other studies have also reported increases in the levels of PA in various intervention trials within a range of LBP populations employing questionnaire-based measurements of PA [43,44]. As all participants in the current study were undergoing physiotherapy interventions during the data collection period, it is not known whether the lack of a change in activity is due to a selection bias, in that participants were already motivated and relatively active at baseline (Table 4), and thus levels of reported activity were relatively high [45]; or that these interventions did not result in a change in the participant’s PA behaviour at 3 months.

Although the majority of participants reported that they had not returned to “normal” activity levels at baseline, it is possible that their actual levels of activity may have been relatively “normal”. Results showed that baseline activity was not correlated with LBP disability or pain levels (Table 8), and that a report of ‘normal’ activities was not correlated with activity levels at the two time points (Table 9). We therefore investigated the possibility that those participants with lower levels of activity at baseline (as recorded with the RT3 and 7D-PAR), might have greater potential to change their activity, and thus to more likely show a relationship with RMDQ at 3 months. Although those with lower activity at baseline had significantly greater change in activity (data not shown) no predictive relationship with RMDQ or change in RMDQ at 3 months was shown. Change in activity was not a predictor of recovery in those participants with lower activity levels at baseline in this study group. However, it is also acknowledged that the current choice for a low activity level chosen (below the mean value for both RT3 VM/hr/week and PAEE (kcals/kg) represented an arbitrary and pragmatic choice and potentially further exploration of activity change within lower baseline activity levels is warranted.

The multivariate model also included reported activity levels prior to the onset of LBP as potential confounders to the relationship between PA and RMDQ; however it is also acknowledged that there were not sufficient numbers to investigate potential interaction effects between prior activity levels and activity levels during the episode of LBP and levels of LBP disability. Also, the percentage in manual occupations (44%) was relatively high, and the fact that the vast majority continued to work was undoubtedly a factor in the high activity levels at baseline [46]. Therefore, the lack of observed change in activity from baseline to 3 months in the group may potentially be due to the moderate RMDQ scores at baseline and the fact that the majority continued to work during this episode of LBP; as a consequence these results cannot be generalised to LBP populations with higher levels of disability and in those who are unable to work.

A patient report of a return to full ‘normal’ activities at 3 months was associated with a greater RMDQ change. This finding has not previously been reported, reflecting that the 24 item RMDQ predominantly assesses activity limitations [32], and is better targeted at populations with either moderate or high disability, similar to our group at baseline [47]. A significant proportion at baseline reported that they had not returned to ‘normal’ activities; this finding is similar to previous research which showed that people with LBP feel some degree of limitation in activities of daily living which correlates to their degree of disability [48]. Surprisingly, an early return to “normal” activities was not an independent predictor of either disability or change in disability at 3 months. The reason for this finding is unknown however, it may relate to the complex nature of the disablement process [49] and the interaction with activity and behavioural factors associated with LBP [50]. The effects of PA on disability have been found to be mediated by a range of factors including pain, fatigue, depression, and self- efficacy [51,52], as well as a range of specific performance issues including trunk flexion and extension and hip, knee and foot pain [8]. Further investigation is therefore required to study the potential effects of other mediators in the relationship between activity, disability, and functional limitation.

Interestingly, there was no relationship found at either time point between the patient’s recorded activity levels and a report of a return to full ‘normal’ activities (Table 8). These results suggest that the patient’s perceived activity levels, rather than their actual levels of activity, maybe more important predictors of recovery. Perceived activity decline has previously shown a significant association with disability, fear of injury, depression, and pain intensity [10,53]. The relationship between patients’ report of activity normalization and perceived activity decline therefore warrants further investigation.

Although no predictive relationships were found between measures of fear avoidance, depression and anxiety, and PA levels and levels of disability, several studies have reported behavioural and psychosocial influences on activity change [44,54,55]. Although a recent study found that fear avoidance did not alter the relationship between activity and fitness [56]. The fear avoidance scores reported in the current study (Table 2) are not of a proposed magnitude likely to increase the risk of chronicity [57], which may help to explain the initial high PA levels. Therefore, it appears that any relationship between activity and fear avoidance and disability is complex; dependent upon both participant activity levels and fear avoidance beliefs.

### Research considerations

The setting and choice of the MCID for the RMDQ is open to some debate. Previous research has demonstrated that baseline RMDQ scores to be relatively high in an ALBP population [58], and taking into account that a 30% change in score has recently been proposed as a meaningful change score for the RMDQ [42] we therefore conservatively set the MCID as a score of 4. Although there was a non-significant change in activity levels over this timeframe it is not known whether the choice of a smaller effect size (MCID < 4) and therefore a larger sample size requirement would have altered the observed results.

The units of activity employed in this research (RT3 VM/hr/wk), although extensively employed in field research as a measure of activity, have not been fully investigated for validity and responsiveness to change in free living research, an essential component of functional activity measures [59]. However, there is no acknowledged and standardised field research protocol for accelerometry use, and few instruments have been evaluated for their ability to reliability evaluate change in activity over time within LBP populations, which remains a weakness of PA measures within populations with disability [60]. Also, the current research employed a number of statistical models to analyse potential predictive relationships within the data sets (n = 83) and acknowledge that although the study was powered to assess such predictive relationships and suitable adjusted analyses performed there is a potential that over fitting of the statistical models may have occurred.

The report of a return to ‘normal’ activities was a dichotomous questionnaire variable and there is scope for a degree of subjectivity as to what patients perceived as their normal level of activity. Further exploration of the perception and return to ‘normal’ activities is warranted based on the findings from the current study.

The current study did not measure the type of physiotherapy intervention that the patient received, and how this might have influenced the patient’s activity and recovery; however, it was presumed that all therapists were adhering to physical activity guidelines in respect to the advice given to patients. Although recommendation exist for evidence based physiotherapy management of acute LBP [61], research demonstrates that there is much variability in the primary care management of LBP [62] in that not all primary care health practitioners adhere to current best practice guidelines. Therefore, further research is required to investigate whether specific therapeutic interventions have a greater effect on a patient’s activities during an episode of acute LBP

There is mixed evidence for activity behavior to change when being monitored [63,64], and higher levels of activity recorded at baseline may be a result of the novelty of activity measurement. The two measurement points chosen mean that activity changes may have occurred outside this temporal window, perhaps as a result of treatment, and thus activity levels may have regressed to a mean level of activity by 3 months. A greater number of measurement points, coupled with the use of other activity measurement tools offer further opportunities to study the relationship between activity and LBP outcomes.

The strength and validity of the findings from this study are supported by powered sample size to detect a MCID in the RMDQ, coupled with the relatively high participant response rate at 3 months, and low levels of RT3 data loss. RT3 data loss was predominately due to RT3 factors such as malfunction and RT3 loss rather than participant factors, which means that data are likely to be completely missing at random and therefore unlikely to have caused any systematic bias in the results.

## Conclusion

This study is the first to prospectively follow an acute LBP population and investigate how activity levels change over time, and whether activity levels predict recovery. Results showed that physical activity levels at baseline and change in activity from baseline to 3 month within this acute LBP population was not a predictor of disability or change in disability. There was also no overall difference in the mean levels of activity as recorded by the RT3, or types of activity from the activity questionnaire and activity diary at the two time points. These results question a possible assumption that activity levels change as LBP symptoms resolve, and also the potential role that physical activity plays in LBP recovery. The patient’s self-report of a return to full ‘normal’ activities was associated with improved functional recovery, and therefore a focus on activity normalisation, rather than specifically increasing activity, may offer the best opportunity for success in improving patient outcomes.

## Abbreviations

ACC: Accident Compensation Corporation; ALBP: Acute low back pain; BPAQ: Baecke Physical Activity Questionnaire Fear-Avoidance Beliefs Questionnaire; EE: Energy expenditure; GHQ12: 12-item General Health Questionnaire; LBP: Low back pain; MCID: Minimal Clinically Important Difference; OR: Odds ratio; PA: Physical activity; PAEE: Physical activity energy expenditure; PI: Principal investigator; RMDQ: Roland Morris Disability Questionnaire; VAS: Visual Analogue Scale; FABQ: Fear Avoidance Beliefs Questionnaire; TDEE: total daily energy expenditure; VM: Vector magnitude; ΔBPAQ: change in Baecke Physical Activity Questionnaire; ΔRT3: Change in RT3 VM/hr/wk; TDEE: Total Daily Energy Expenditure; PAEE: Physical Activity Energy Expenditure change in RT3 VM; ΔRMDQ: change in Roland Morris Disability Questionnaire score; 7D-PAR: 7-Day Recall Questionnaire.

## Competing interests

All authors declare that they have no competing interests.

## Authors’ contributions

PH is the principal investigator. PH together with his supervisory team of SM, MB, LH and DB designed the study and were responsible for the protocol. SMc and DH acted as international advisors and helped in the development of key ideas underlying this study. PHerbison was responsible for the statistical analysis and the evaluation of the database. All authors contributed to the writing of the manuscript.

## Pre-publication history

The pre-publication history for this paper can be accessed here:

http://www.biomedcentral.com/1471-2474/14/126/prepub
